# Amphiphilic Histidine-Based Oligopeptides Exhibit pH-Reversible Fibril Formation

**DOI:** 10.1021/acsmacrolett.1c00142

**Published:** 2021-07-15

**Authors:** Carlos Noble Jesus, Rhys Evans, Joe Forth, Carolina Estarellas, Francesco Luigi Gervasio, Giuseppe Battaglia

**Affiliations:** † Department of Chemistry, University College London, London WC1H 0AJ, United Kingdom; ‡ Institute for the Physics of the Living System, University College London, London WC1E 6BT, United Kingdom; § Pharmaceutical Sciences, University of Geneva, 1211 Geneva, Switzerland; ∥ Institute for Bioengineering for Catalonia, The Barcelona Institute for Science and Technology, 08028 Barcelona, Spain; ⊥ Catalan Institution for Research and Advanced Studies (ICREA), Barcelona, Spain

## Abstract

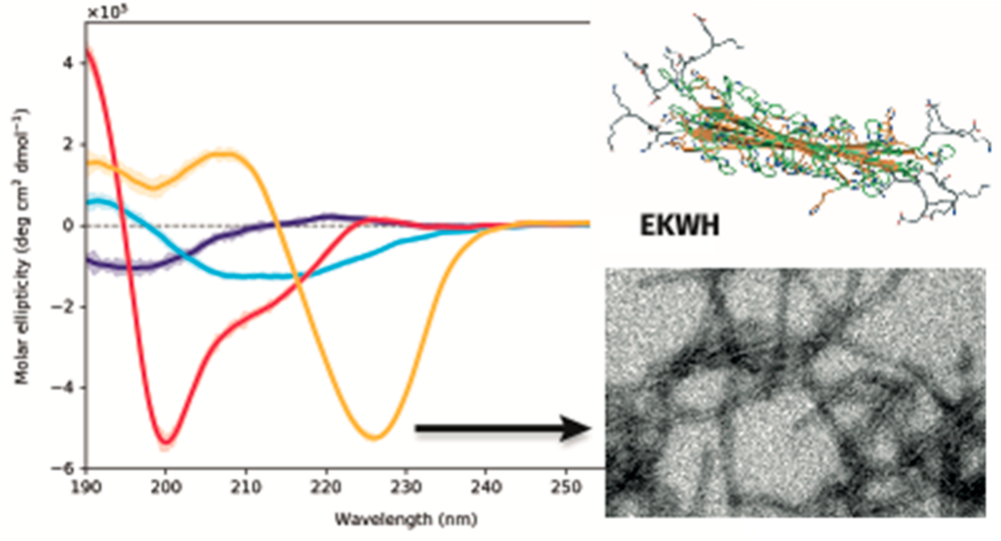

We report the design, simulation, synthesis, and reversible self-assembly of nanofibrils using polyhistidine-based oligopeptides. The inclusion of aromatic amino acids in the histidine block produces distinct antiparallel β-strands that lead to the formation of amyloid-like fibrils. The structures undergo self-assembly in response to a change in pH. This creates the potential to produce well-defined fibrils for biotechnological and biomedical applications that are pH-responsive in a physiologically relevant range.

Peptides are biocompatible as well as biodegradable and can be readily synthesized with high sequence complexity and low polydispersity by using a range of methods. Additionally, ensembles of peptides can undergo self-assembly into nanostructures with complex structure–function relationships including pores and fibrils as well as many more exotic systems.^1,2^ Exploiting these properties has led to applications in disciplines ranging from nanomedicine to solid-state physics;^3,4^ however, achieving controlled self-assembly of peptides is often challenging. Traditional molecular engineering efforts typically rely heavily on extant structural motifs in biology,^5^ incorporating a non-peptide element to simplify supramolecular interactions^6,7^ or studying simple di- or tripeptide sequences.^8^ Several peptide systems have used pH-responsiveness to help control the self-assembly process by allowing the secondary structure of the peptide sequence in different pH ranges to dictate the final tertiary and quaternary structures.^9−11^ More recently, combinatorial and evolutionary approaches, both experimental and *in silico*,^12,13^ have been used to successfully engineer supramolecular peptide assemblies that have proven useful in applications including hydrogel design and emulsification.^14,15^ However, studies of larger oligopeptides remain beyond the reach of computational approaches, while dynamic combinatorial library approaches often provide limited fundamental insight. Consequently, examples of the engineered self-assembly of ensembles of synthetic oligopeptides into structures with controlled dimensionality, structure, and function remain both desirable and scarce.

Amyloid fibrils are a particularly common structure among β-sheet forming peptides,^16,17^ often found in several pathologies including amyloidosis, Parkinson’s disease, and Alzheimer’s disease. However, amyloid fibrils also perform physiological functions in organisms ranging from prokaryotes to humans,^18^ such as in pigmentation, peptide hormone storage, and modulating antimicrobial response.^19^ The formation of fibrils usually occurs due to the aggregation of β-structures to produce a more energetically favorable configuration. The cross-β-sheet rich structure bestows fibrils with high stability and tensile strength.^16,20,21^ These unique properties have led many to take advantage of them in bioprinting, in controlled release, or as a matrix for cell adhesion.^22−24^ By harnessing their innate properties, we can leverage the functional, nonpathogenic side of amyloid fibril structures for biotechnological and biomedical applications.

Herein, we report the rational synthesis and self-assembly of short pH-sensitive histidine-based amphiphilic oligopeptides that produce amyloid fibril structures. By combining the physiologically relevant pH-sensitivity of histidine with the inherent propensity of aromatic amino acids to promote π–π stacking and hydrophobic interactions, we have created unique peptides with highly controllable self-assembly. This constitutes the first histidine-based fibrils that are capable of reversible assembly in response to a physiologically relevant stimulus, opening up the possibility for use in biological applications.

The design for our histidine-based peptide revolved around a diblock copeptide sequence that would be amphiphilic at neutral pH and possess antifouling properties: (EK)_2_-(H)_12_. Histidine was chosen for the hydrophobic block as its protonated imidazolium side group has a p*K*_a_ of ∼6.0, similar to the acidic pH found in endosomes,^25^ allowing for reversible self-assembly of the structures at a physiologically relevant pH. The hydrophilic block was composed of alternating units of glutamic acid (E) and lysine (K) to produce a zwitterionic block with a net charge of ∼0.

We created three further histidine-based amphiphilic oligopeptides with the addition of three different hydrophobic amino acids in the histidine block. The purpose of these additions was to (a) determine whether their presence affected the pH-sensitivity of the block, (b) assess their effect upon the hydrophobicity of the histidine block, and (c) understand whether their incorporation into the histidine blocks would affect the secondary structure and, hence, fibril formation. We selected one nonaromatic amino acid, isoleucine (I), and two aromatic amino acids, phenylalanine (F) and tryptophan (W), to produce (EK)_2_-(IH)_6_, (EK)_2_-(FH)_6_, and (EK)_2_-(WH)_5_, respectively. We refer to these four peptides collectively as EKXH peptides and as EKH, EKIH, EKFH, and EKWH, respectively, when referring to the individual peptides. Of the hydrophobic amino acids, the use of phenylalanine in the formation of fibrils is especially well-documented, with several examples of Fmoc-FF nanotubes and nanofibrils in the literature.^26−28^

Our EKXH peptides were synthesized by using standard Merrifield solid-phase peptide synthesis and purified by preparative HPLC. The accuracy of the peptide sequences was determined from their molecular weights by using MALDI-TOF. Before self-assembly, the lyophilized peptides were first dissolved in pH 4 PBS solution. The peptides were then self-assembled by using the pH switch technique, whereby the pH of the peptide solution is steadily increased by using a syringe pump to inject 1 M NaOH at a rate of 1 μL/min. The pH switch was performed until the final pH of each peptide solution was pH 7.4. During the self-assembly process, the peptide solutions transitioned from transparent to turbid in appearance (Figure 1). The pH-sensitivity of the peptides was quantitatively determined by using turbidity assays (Figure S1), demonstrating that the self-assembly of the EHXH peptides was pH-reversible and was governed by the deprotonation of the imidazole group in all cases.

**Figure 1:**
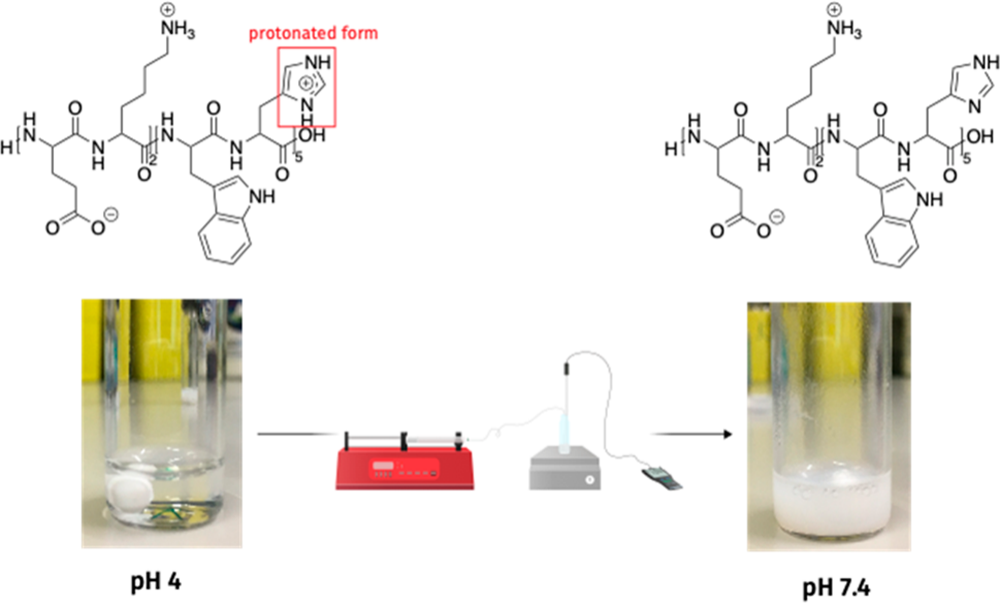
Self-assembly of the peptides from pH 4 to pH 7.4 using the pH switch method. The initial clear peptide solution becomes turbid as the pH increases, indicating the formation of insoluble particles.

After self-assembly, the peptide samples were left to stand to observe their colloidal stability; within minutes, sedimentation of the peptide structures occurred (Figure S2a). The peptides could be redispersed by agitating the sample, but they subsequently sedimented again. Autocorrelation functions from DLS measurements (Figure S2b) showed the presence of very large structures (micrometer sized or larger).

Circular dichroism (CD) measurements were taken of the EKXH peptides at pH 7.4 following self-assembly in water to compare the effect of the addition of different hydrophobic amino acids to the histidine block (Figure 2a).

**Figure 2:**
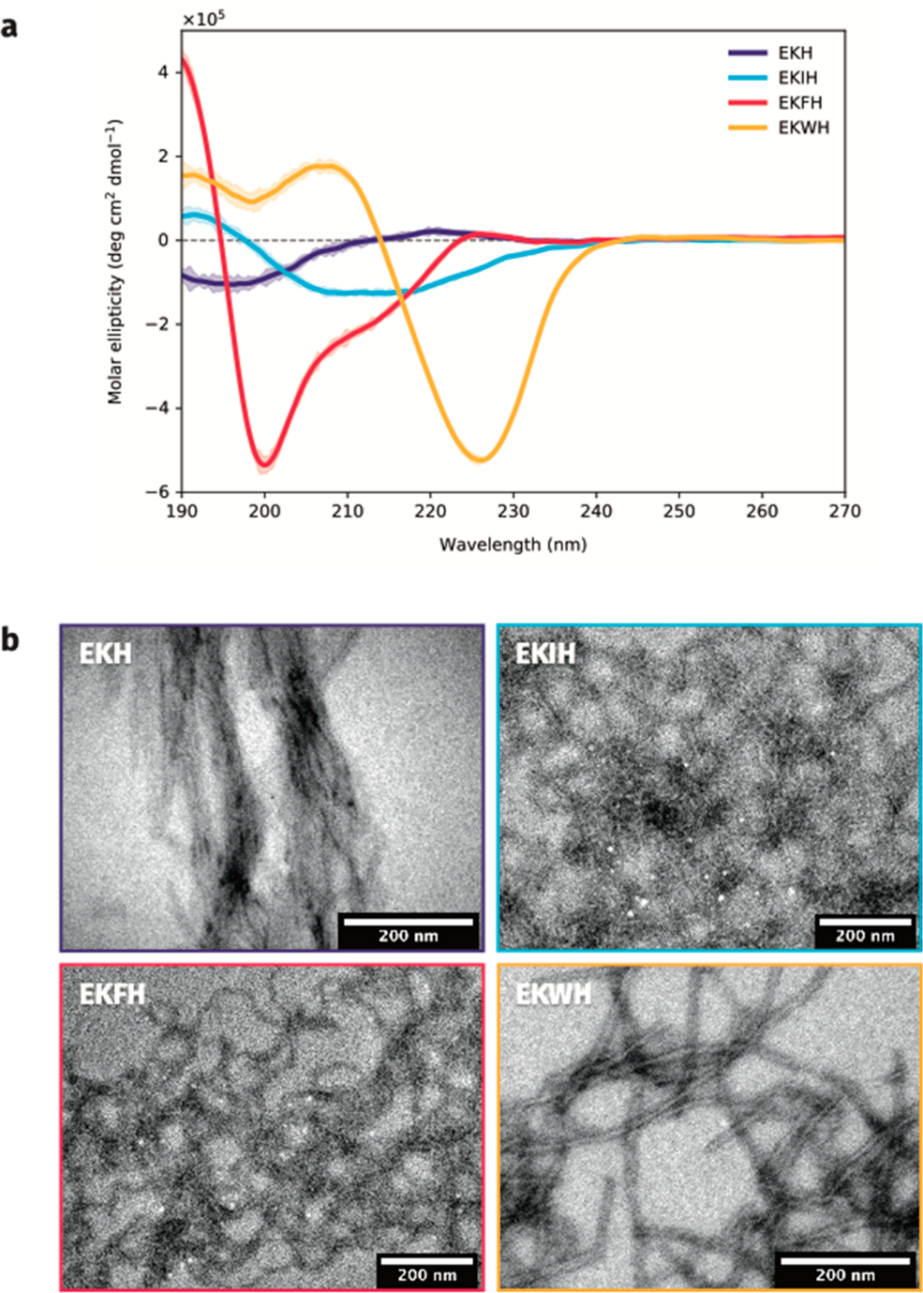
(a) Circular dichroism spectra of the EKXH peptides. EKH is included as a baseline to demonstrate the change in secondary structure that occurs when adding different hydrophobic amino acids to the peptide sequence. (b) Transmission electron microscopy images of the self-assembled structures for EKH, EKIH, EKFH, and EKWH.

The CD spectrum of EKH showed that the peptide adopted a random coil conformation, with a characteristic wide negative band at around 200 nm.^29^ In comparison, CD spectra for the remaining peptides with aromatic amino acids clearly showed the presence of antiparallel β-strands,^30^ suggesting that the inclusion of aromatic amino acids in the histidine block promoted the β-strand secondary structure. The distinct spectroscopic signatures produced by EKFH and EKWH indicated the formation of right-hand-twisted and left-hand-twisted antiparallel β-strands, with sharp minima of great magnitudes at approximately 200 and 225 nm, respectively.^30^

TEM images of the self-assembled EKXH peptides showed fibrillar structures for all three peptides (Figure 2b), with EKWH exhibiting the most well-defined fibril structures. The fibrils for all the peptides had a width of ∼10 nm, with lengths of micrometers or more. Together, the CD and TEM results suggest that the inclusion of aromatic amino acids has a profound effect on creating ordered structures. In particular, EKWH showed a very well-defined β-sheet structure, suggesting the formation of amyloid-like fibrils, which are composed from a predominantly β-sheet structure that produces a cross-β conformation.^16^

Upon closer inspection, high-resolution TEM imaging of EKWH showed the formation of extended fibrils with a typical length of 0.5 μm or more, widths between 10 and 40 μm, and a lamellar structure with a repeat distance of 35 ± 5 Å (Figure 3a–c and Figure S3). The dark bands in the micrographs are a result of the samples being stained with phosphotungstic acid (PTA), which binds more strongly to carboxylic acid groups than carbonyl groups,^31^ corresponding to the locations of the glutamic acid groups in the hydrophilic moiety as well as the C-termini of the peptides. The characteristic spacing of the dark bands is attributed to EKWH arranging itself linearly, with the N-terminus of one peptide next to the C-terminus of another.

**Figure 3:**
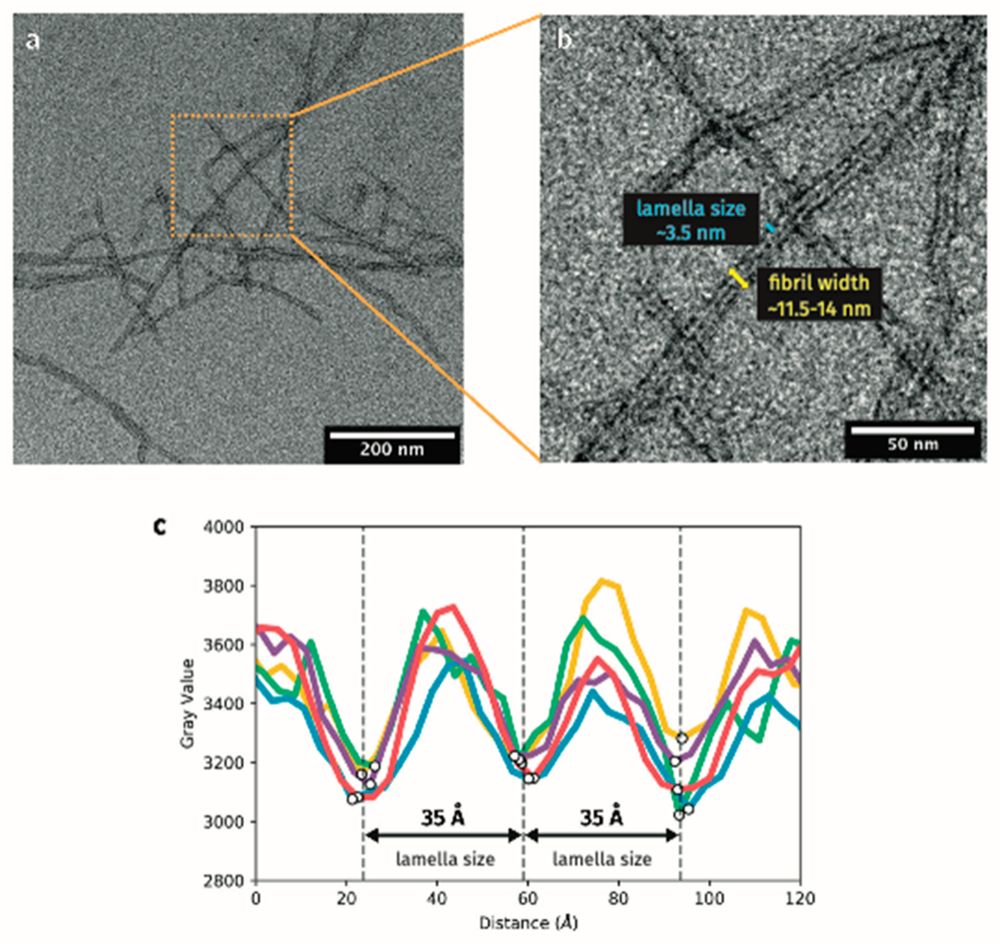
(a, b) TEM micrographs showing lamellar structure in EKWH fibrils. (c) Measuring the separation between the dark bands in the fibrils yields a repeat distance of ∼35 Å.

Simulations were employed to investigate the structure and behavior of the target peptides at an atomistic level. Each peptide (EKH, EKWH, EKIH, and EKFH) was initially constructed by using AmberTools *tleap* and *cpptraj* (Figure S4).^32^ The standard protonation state at physiological pH was assigned to the ionizable residues, with special consideration for the histidine residues, which were set as monoprotonated. To corroborate the CD results, the β-strands were built in both parallel and antiparallel arrangements (Φ = −119°, Ψ = 113°) and (Φ = −139°, Ψ = 135°) for parallel and antiparallel, respectively (Figure S4a).^33^ Six of these individual β-strands were then arranged into β-sheets by using a custom PyMOL script,^34^ with a defined inter-peptide C_α_–C_α_ distance of 4.9 Å. Two possible arrangements of the strands were considered for the antiparallel β-sheets: a same-sided arrangement, in which all the histidine side chains were located on the same side of the β-sheet, and an alternating arrangement, where both His and Ile, Phe, or Trp side-chains were expressed on both sides of the β-sheet in an alternating fashion (see Scheme S1 and Figure S4b in the Supporting Information for details). Finally, lamellar structures were constructed from three of these antiparallel β-sheets in their rigid and unequilibrated form, with an intersheet distance of 11.5 Å to avoid clashes between the extended side-chains (Figure S4c). To maintain a balance between computational cost and accuracy, lamellar structures made from three β-sheets were studied to test the behavior of the internal and external interactions of the much larger fibrils. In these lamellae, the distance between the two areas within the peptide with carboxyl groups (the C-terminus and the region with the glutamic acid residues) was found to be ∼35 Å. This was in excellent agreement with the lamellae repeat distances observed via TEM.

All-atom, explicit solvent simulations were performed by using GROMACS 2018.3^35^ with the a99SB-*disp* force field,^36^ which has been shown to provide accurate results, specifically when focusing on intrinsically disordered proteins analogous to the peptides under investigation here.^37^ See the Computational Methods section in the Supporting Information for details about the building of the systems and the simulations performed. The final structures from the equilibration process were used as the starting point for the molecular dynamics (MD) simulations. Each simulation was run for 1 μs for all of the systems. Figure 4 shows the time evolution of the root-mean-square deviation (RMSD) from the initial, *ideal* structures throughout each of the alternating antiparallel β-sheet simulations, which is used as a direct measure of the β-sheets’ stability. All the systems start from a similar position, but quickly EKIH, EKFH, and EKH, to a lesser extent, undergo a conformational change. On the contrary, EKWH settles into a stable natural β-sheet arrangement. We have also clustered the structures exhibited during the last 500 ns of the MD simulations (see Histograms in Figure 4). For EKWH and EKFH, the majority of the simulation snapshots are characterized in the first cluster, representing 79% and 66% of all the structures explored during the simulation, respectively. In these cases, the difference between the first cluster and the second and third clusters is very significant, as these subsequent clusters contain far fewer structures than the first. Contrastingly, we would like to emphasize the significant difference in the behavior observed for EKH and EKIH. In the former, the first three clusters, which account for the highest representation of the structures, were composed of only 16% of the structures in the first cluster down to the 9% in the third cluster. EKIH, potentially due to the introduction of a nonaromatic amino acid, produced an even greater distribution of structures, with the first three clusters only including 6% to 3% of the simulated structures. This means that the fluctuation shown by this system along the dynamics is very high, and it is an indication of unstable structure. Additionally, Figure 4 shows a representative snapshot of the first cluster for each of the systems, further demonstrating that EKIH evolved to a disordered structure and EKH saw significant distortion of one of the β-strands, while EKFH and especially EKWH were very stable, maintaining their structure for the full 1 μs. These results were in agreement with the experimental results (further results, including those for the parallel β-sheets, can be found in the Supporting Information, Figure S5).

**Figure 4:**
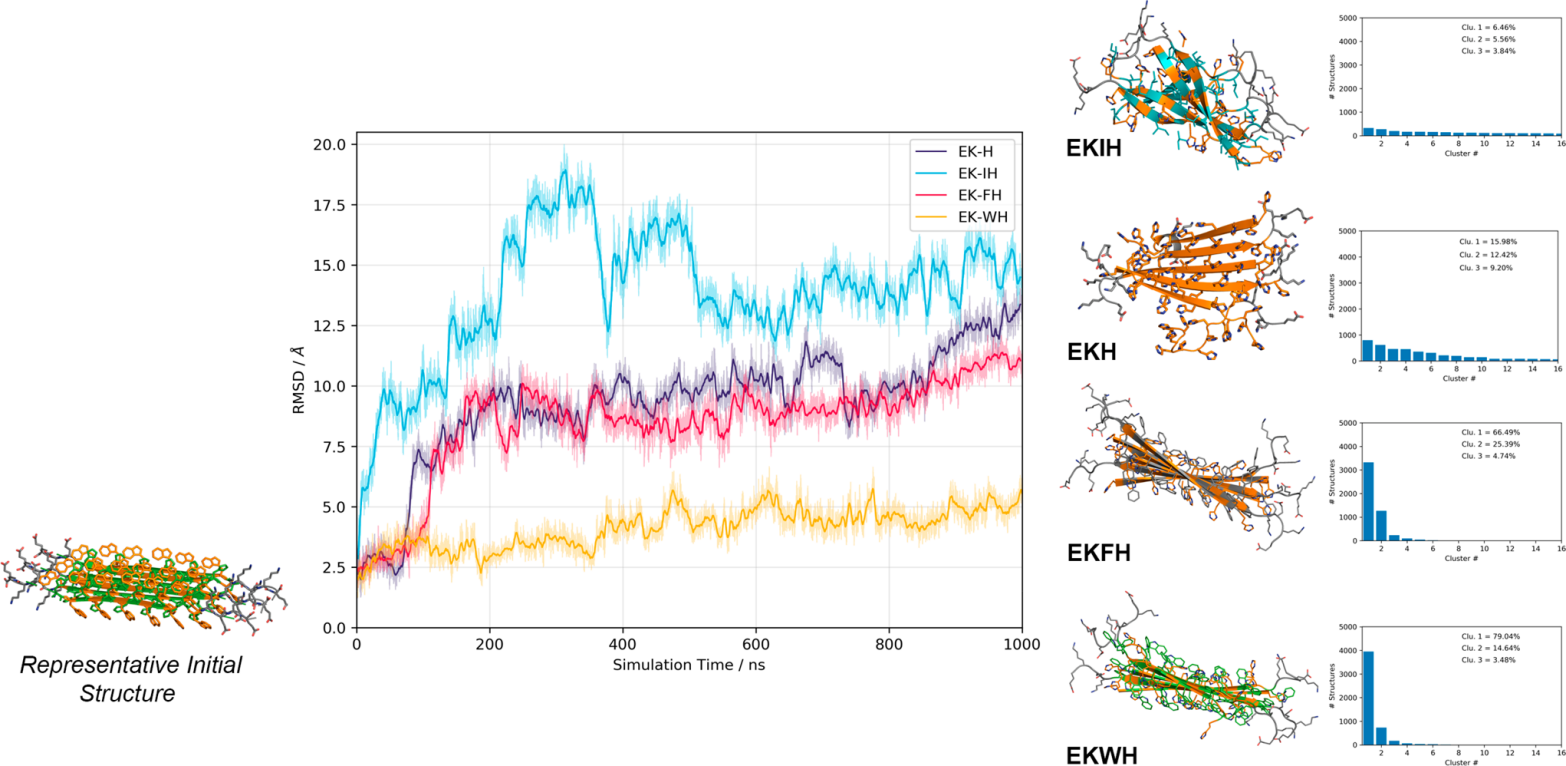
Time evolution of the root-mean-square deviation (RSMD, Å) of the alternating antiparallel β-sheet structures for the MD simulations of EKH (purple), EKIH (cyan), EKFH (magenta), and EKWH (orange). Comparison between the representative initial structure (left) and stabilized structures (right) visually shows the conformational changes of each β-sheet during the MD simulations. Representative snapshots were clustered for the last 500 ns of the simulations, the results of which are shown in the histograms (far right).

Analysis of the root-mean-square fluctuation (RMSF) per residue (Figure S6) demonstrated an increase in stiffness from EKIH < EKH < EKFH < EKWH, in both the parallel and antiparallel arrangements. However, the overall stabilization of the antiparallel β-sheets was more significant. These measurements of the systems match the observed rigidity and stability scaling of the experimental results.

In light of both the experimental and simulated results for the β-sheets, we only considered the most unstable (EKIH) and most stable (EKWH) β-sheets when building the lamellar structures. Figure S7a shows the evolution of the RMSD along the 1 μs lamellae simulations, indicating that EKIH undergoes an important change, deviating from the initial structure during the first 400 ns, and then is stabilized in a different conformation. For the EKWH lamellae, we performed the simulations for both the same-sided and the alternating antiparallel arrangements (Figure S4b) to provide a better contrast of intersheet interactions. While the same-sided EKWH lamellae suffer a significant destabilization in the first 500 ns, only to then stabilize in a disordered conformation, the alternating arrangement demonstrates a stable behavior for the whole 1 μs simulation. These results were also confirmed by cluster analysis and a subsequent evaluation of the hydrogen bond interactions within the lamellar structures (see Figure S7b–d). The RMSF analysis (see Figure S8) corroborates these conclusions. It highlights that for the alternating EKWH lamellae the only mobile residues are in the hydrophilic blocks, while for EKIH and the same-sided EKWH there is higher motion in the hydrophobic blocks of peptides in the outer sheets. These results have been confirmed by the analysis of contact maps between initial and final snapshots of all the lamellar systems under consideration. We have evaluated which interactions changed the most along the simulation, both within and between the β-sheets, and have demonstrated how changes in the distribution of interactions directly affect the conformation and stabilization of the lamellar structures (see the Supporting Information for further discussion, Figures S9–S11). In summary, the differences in the interaction network completely change the geometry of the EKIH lamellar structure, while for EKWH the lamellar structure is more stable and maintained along the simulations. These results directly support and allow us to understand the experimental findings.

In summary, we have shown that the insertion of the aromatic amino acids phenylalanine and tryptophan into a short histidine block promotes the reversible formation of well-defined β-sheets and, hence, pH-responsive fibrils. It is particularly remarkable that we can introduce these aromatic amino acids into our amphiphilic oligopeptides, without sacrificing the unique pH-sensitivity bestowed by the histidine residues. Circular dichroism and transmission electron microscopy showed the formation of fibrillar, lamellar structures of tunable mechanical properties. Simulations provided molecular insight into the effect of systematic residue substitutions upon secondary structure and self-assembly. Systematically producing β-sheet structures for use in applications such as tissue engineering and bioprinting can be a difficult challenge. However, our findings demonstrate that we can influence the secondary structure in a well-defined manner by introducing aromatic amino acids into a histidine-based peptide sequence. Furthermore, we have successfully created amphiphilic oligopeptide structures that are pH-sensitive in a physiologically relevant pH range, allowing for controlled assembly and disassembly for biological applications. It is our hope that this insight into the connection between primary and secondary structure with self-assembly may help to further elucidate the mechanisms underpinning peptide assembly, thus allowing for the creation of more complex and versatile next-generation biomaterials.

## Supporting Information Available

Additional results for turbidity experimental assays; computational methods and results of stability of β-sheets and lamellar structures (PDF)
