# Supplementary material for: Amphiphilic Histidine-Based Oligopeptides Exhibit pH-Reversible Fibril Formation
Source: ACS Macro Lett. 2021 Jul 15;10(8):984–9. doi: 10.1021/acsmacrolett.1c00142 (PMC8375021; doi:10.1021/acsmacrolett.1c00142)
Supplement: Supplementary file 1 — mz1c00142_si_001.pdf [file mz1c00142_si_001.pdf]

Supporting Information

Amphiphilic histidine-based oligopeptides exhibit pH-reversible fibril formation

Carlos Noble Jesus[1,2], Rhys Evans[1], Joe Forth[1,2], Carolina Estarellas[1], Francesco Luigi Gervasio[1,3]*, Giuseppe Battaglia[1,2,4,5]*

[1]Department of Chemistry, University College London, London WC1H 0AJ, United Kingdom

[2]Institute for the Physics of the Living System, University College London, London WC1E 6BT, United Kingdom

[3]Pharmaceutical Sciences, University of Geneva, 1211 Geneva, Switzerland

[4]Institute for Bioengineering for Catalonia, The Barcelona Institute for Science and Technology, 08028 Barcelona, Spain

[5]Catalan Institution for Research and Advanced Studies (ICREA), Barcelona, Spain

Experimental Data S2
Computational Methods S5
Computational results obtained for β-sheets S9
Computational results obtained for lamellar structures S12

Experimental Data

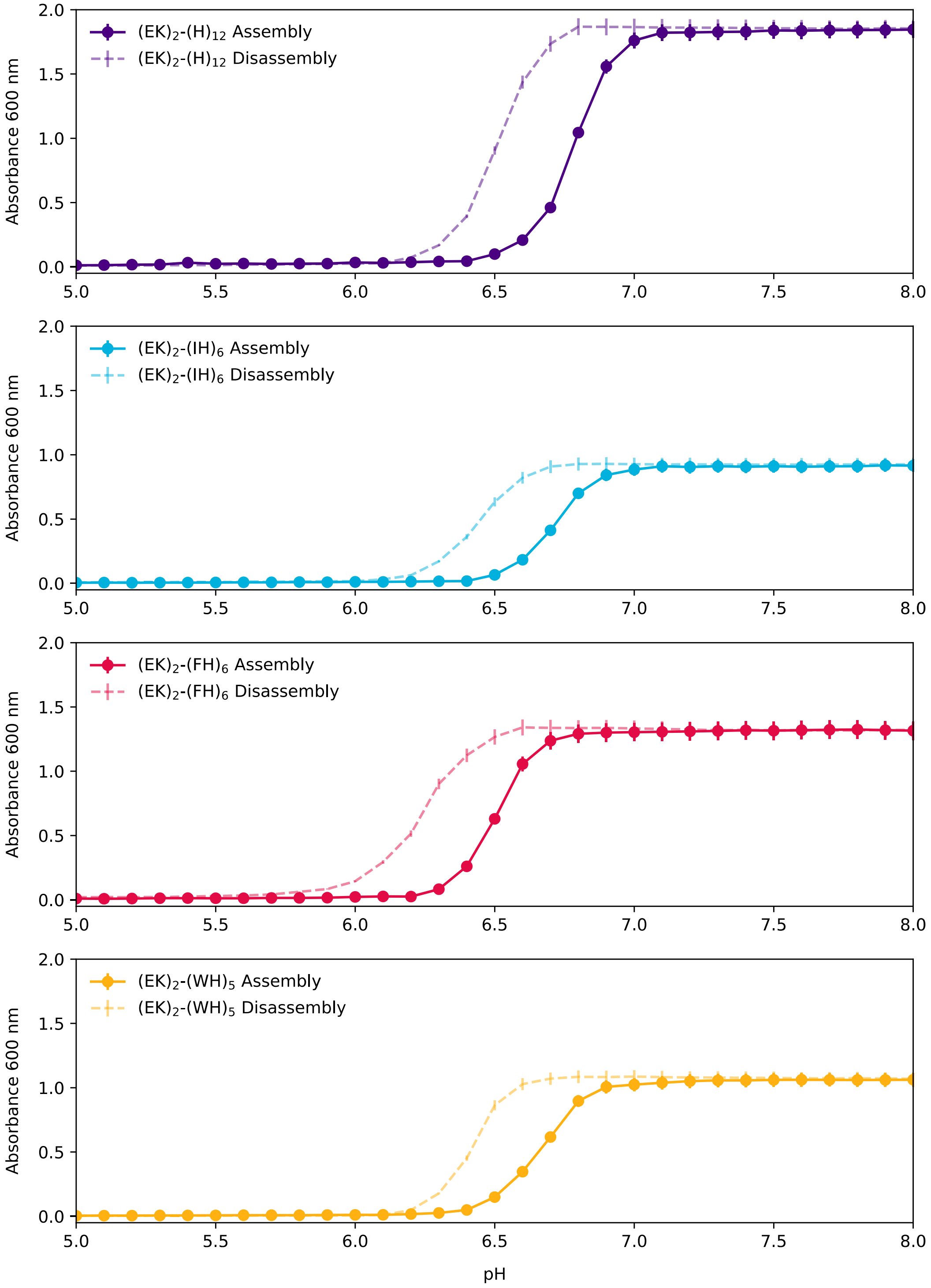

Figure S1. *Turbidity assays of EKXH peptides. The absorbance at 600 nm was measured at multiple points during a pH switch to quantitatively determine the pKa of the peptide, i.e. the point at which self-assembly occurs. Measurements were also taken for disassembly by performing a reverse pH switch, i.e. the addition of HCl. Error bars denote ± 1 standard deviation from three measurements.*

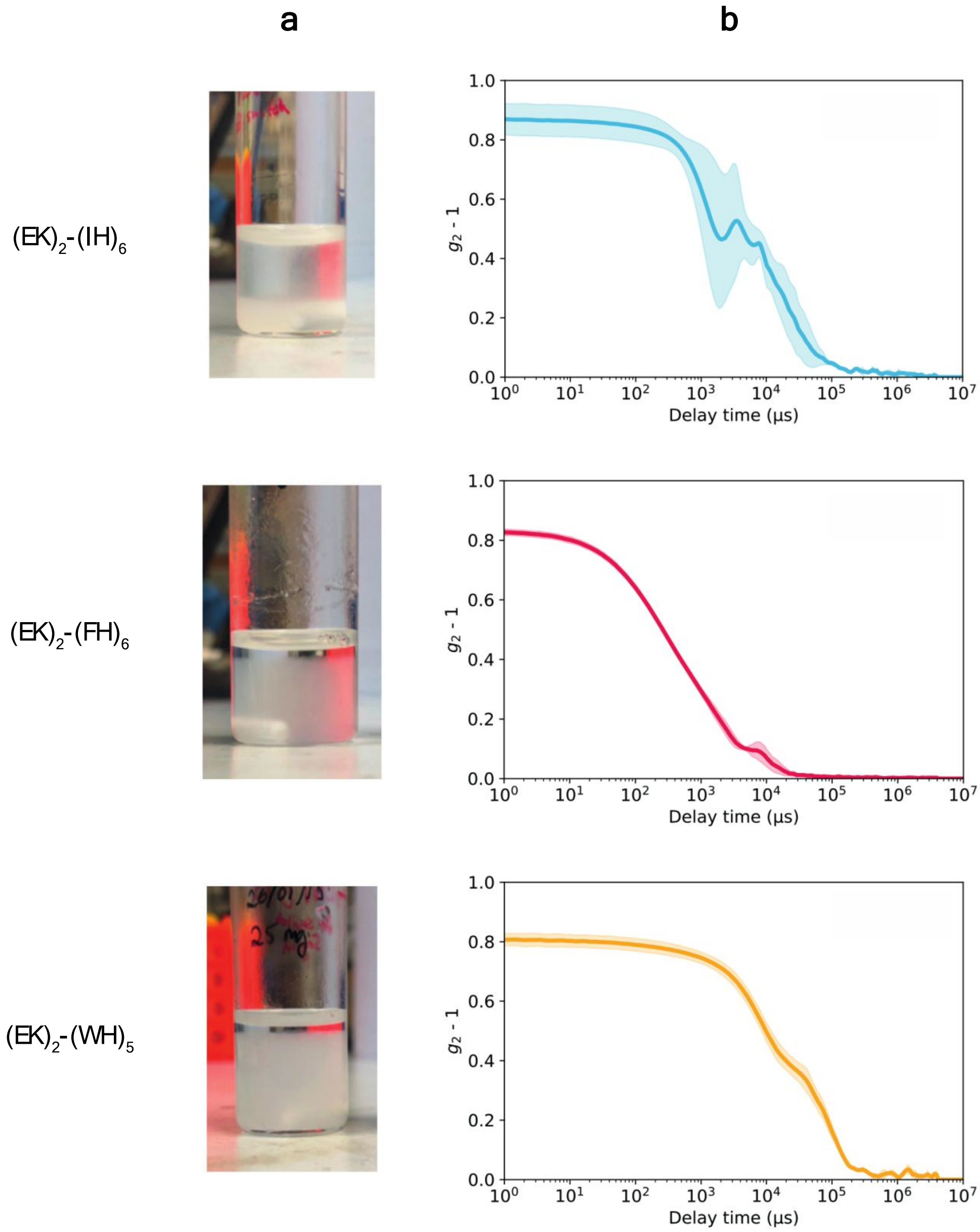

Figure S2. *Large structures with low colloidal stability form during peptide self-assembly. (a) Sedimentation of the EKXH peptides occurs within 1 hour of self-assembly. (b) Auto-correlation functions with large delay times before a non-monotonic decay indicate the formation of very large structures.*

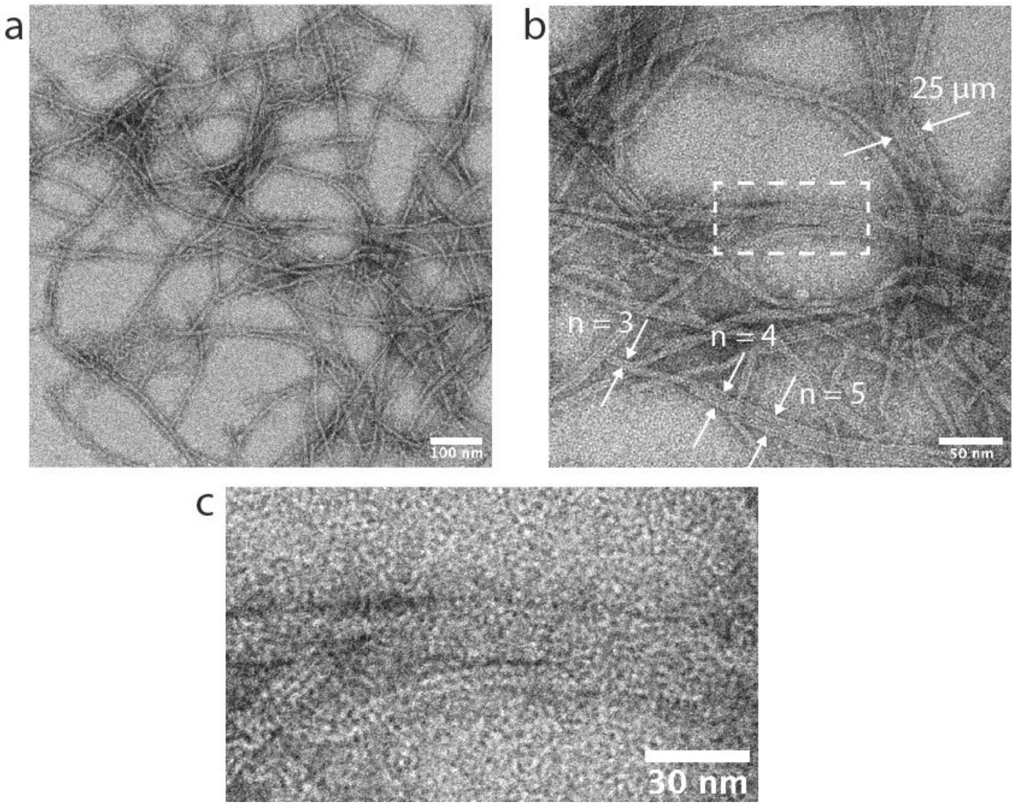

Figure S3. *TEM micrographs showing structures formed by EKWH peptides. (a) Fibril lengths range from hundreds of nanometers up to in excess of microns. (b) Fibril widths vary from 7 nm up to tens of nanometers, and are present in both odd and even numbers of lamellae. (c) Enlargement of the region denoted by the dashed square in Fig. S2b, showing ranching of fibrils.*

Computational Methods

The initial systems were immersed in a pre-equilibrated octahedral box using the four-point water model from the a99SB-disp force field [1], which is a modified version of TIP4P-D. The systems are set-up such that the amino acids are in the appropriate charged states at physiological pH (7.4). Lysine is positively charged, glutamic acid is negatively charged, and the hydrophobic amino acids, tryptophan, isoleucine, and phenylalanine are all neutral. Also for this reason, the histidines were set to be mono-protonated.

They were subsequently minimized using 50000 cycles of steepest-descent energy minimization. The equilibration process was performed in three steps. The first step involved the heating of the system from 0 to 300 K in 1 ns (NVT ensemble) and was followed by two steps of 10 ns equilibration (NPT ensemble) using the velocity-rescale thermostat [2]. In the first of these steps, a Berendsen barostat was used, and the position of the peptides was restrained. The second saw a full relaxation of the system using Parrinello-Rahman pressure coupling. All production runs of MD used periodic boundary conditions and an NVT ensemble. The Particle Mesh Ewald method was used for treating long-range electrostatics using a cut-off of 12 Å [3]. A time step of 2 fs was used for all simulations after imposing constraints on the hydrogen stretching modes. Simulations of the parallel-arrangement systems consisted of approximately 65,000 atoms, while the antiparallel systems required 90,000 atoms due to the extra extension of the hydrophilic blocks on one side. For the lamellar systems, over 110,000 total atoms were used in the simulation box.

Alternating vs. Same-sided β-sheet Construction

Due to the alternating sequence of the hydrophobic residues, when each peptide is constructed in a β-strand conformation, it results in the side chains of the same residue having the same orientation. For example, all the histidine residues will be facing *up* in relation to the plane of the β-sheet. Consequently, when constructing the β-sheet, there are two options; having all the β-strands in the same orientation, e.g. having all histidines facing *up* on all strands; or having them alternating, a strand with histidines *up* next to one with histidines *down* (see Scheme S1a). We have simulated both options for EKWH β-sheets. This has potentially more important implications for the lamellar structures, within which there would be layers of each type of residue which could create significantly stronger attractive or repulsive interactions (Scheme S1b).

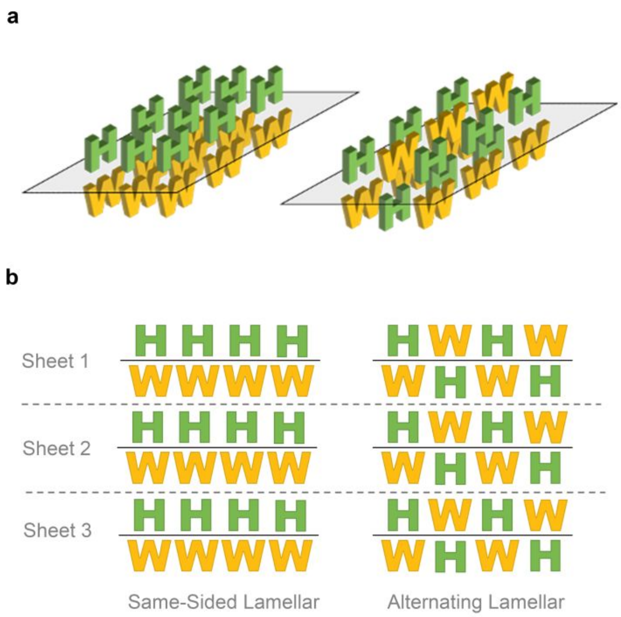

Scheme S1. *Schematic representation of the various arrangements of side-chain orientations used in this investigation. (a) Same-sided (left) and alternating (right) antiparallel β-sheets. (b) Same-sided (left) and alternating (right) antiparallel β-sheets for the construction of the lamellar systems.*

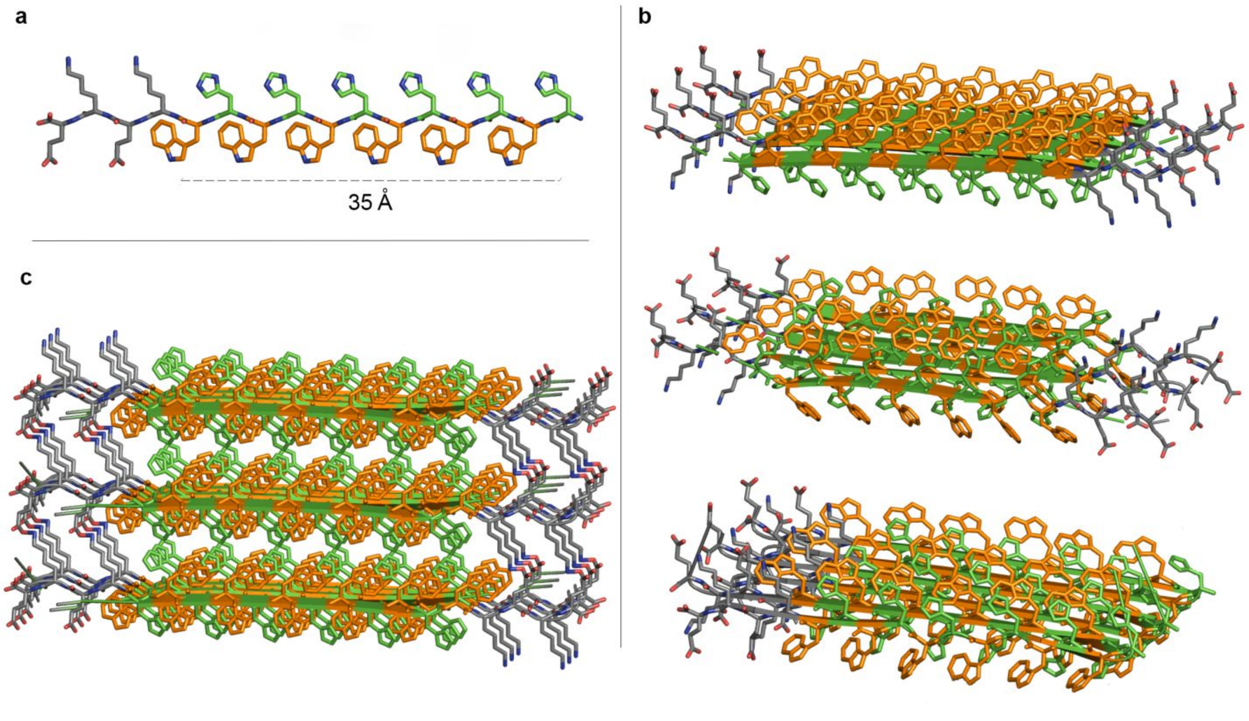

Figure S4. *The EKWH peptide is used to illustrate the different conformations that were simulated. (a) β-strand obtained from the tleap and ccptraj Ambertools. (b) β-sheets constructed in the same-sided antiparallel arrangement (top), alternating antiparallel arrangement (middle), and alternating parallel arrangement (bottom). (c) Lamellar structure constructed from three alternating, antiparallel β-sheets. Each β-sheet is arranged directly on top of the other, with the minimum possible separation that avoided side chain clashes.*

Cluster Analysis

Clustering was performed on the last 500 ns of each simulation using the GROMACS cluster tool with the *gromos* algorithm as described in Daura et al. [4]. After an number of trials with both data sets, an RMSD cut-off of 3 Å was used for the β-sheet simulations and 2.5 Å was selected as the cut-off for the lamellae simulations. The central structure of the most populated cluster was selected as the representative, equilibrated structure of the simulation. These analyses have been carried out for the β-sheets and the lamellar structures.

Contact Maps

Contact maps are used to analyze the network non-bonded interactions between atoms at any given point during the simulation. From an input structure file, you must first identify the total feasible pairs of atoms that will form a contact, based on the type of atom and their separation. For the lamellar structures, this would typically result in a total of around 1000 contacts. Because systems are mobile and non-bonded interactions are by their nature transient and flexible, a switching function is used to determine whether these contacts are formed, instead of a hard cut-off. In this implementation, the switching function is a rational switching function (s) of the distance between the atom pairs (r) and given by:

$$s(r) = \frac{1 - \left(\frac{r - d_0}{r_0}\right)^n}{1 - \left(\frac{r - d_0}{r_0}\right)^m}$$

Where $d_0 = 0.0,\ n = 6,\ m = 2n$. The value of r_0, the expected value for each contact, is optimized for each contact individually. For $r \le r_0$, $s(r) = 1.0$ whilst for any distance larger than r_0 the value of $s(r)$ decays smoothly to 0.0. This provides a range of values and provides leniency as to whether a contact is formed. When comparing two contact maps for the same system, it is these values of the switching function for each contact that we use to evaluate the differences. For contacts that change, i.e. are formed or broken during the simulation, a difference of $|s_{initial} - s_{final}| \geq 0.75$ was used. Typically, for the lamellar systems under consideration in this paper, this reduced the total of 1000 contacts down to 300-400. To define contacts that were definitely maintained throughout the simulation, a cut-off of $|s_{initial} - s_{final}|$ ≤ 0.4 was used.

References

[1] Robustelli, P.; Piana, S.; Shaw, D. E. Developing a Molecular Dynamics Force Field for Both Folded and Disordered Protein States. *Proc Natl Acad Sci USA* **2018**, *115* (21), E4758–E4766. https://doi.org/10.1073/pnas.1800690115.

[2] Bussi, G.; Donadio, D.; Parrinello, M. Canonical Sampling through Velocity Rescaling. *The Journal of Chemical Physics* **2007**, *126* (1), 014101. https://doi.org/10.1063/1.2408420.

[3] Darden, T.; York, D.; Pedersen, L. Particle Mesh Ewald: An $N \cdot \log(N)$ Method for Ewald Sums in Large Systems. *The Journal of Chemical Physics* **1993**, *98* (12), 10089–10092. https://doi.org/10.1063/1.464397.

[4] Daura, X.; Gademann, K.; Jaun, B.; Seebach, D.; van Gunsteren, W. F.; Mark, A. E. Peptide Folding: When Simulation Meets Experiment. *Angewandte Chemie International Edition* **1999**, *38* (1-2), 236–240. https://doi.org/10.1002/(SICI)1521-3773(19990115)38:1/2<236::AID-ANIE236>3.0.CO;2-M.

Computational results obtained for β-sheets

RMSD and RMSF analysis for different β-sheet arrangements

The simulations for the EKWH systems were extended up to 1.5 μs in order to assess the stability between the alternating and the same-sided antiparallel arrangements, showing a clear stabilization of the alternating antiparallel arrangement, most likely due to steric and electrostatic reasons (Figure S5a). For this reason, all the systems were then run in alternating antiparallel and parallel arrangements, respectively. Figure S5sb shows the results for the alternating parallel β-sheets.

Although the trends are, in general, similar to those found in the antiparallel arrangement, with the EKIH being the most unstable one, the difference between EKH, EKFH and EKWH is less significant in this case. In the parallel arrangement, EKWH explored different conformations along the simulation, as is confirmed by the cluster analysis. In this case the first cluster represents 66% of all the snapshot structures, while in the alternating antiparallel it represented 79% of the structures. For EKFH, the difference is even greater, as although the RMSD ends the simulation at a similar value, the cluster analysis reveals a much greater diversity in the structures explored. In this case, the difference between the anti- and parallel structures of the first cluster is significant, decreasing from 66% to 22%, an indication of the instability of the system along the simulation. In the image representing the first cluster of EKFH, it can be observed that one of the β-strands is distorted. The EKH exhibits a similar behavior to EKFH in the RMSD plot, being in between of the two aromatic structures, EKFH and EKWH. However, the representative snapshot of the first cluster, which accounts for 48% of the simulated structures, shows a broken β-sheet. Finally, for EKIH the most unstable and effectively no longer in the β-sheet conformation, although this is a slight improvement over the antiparallel arrangement.

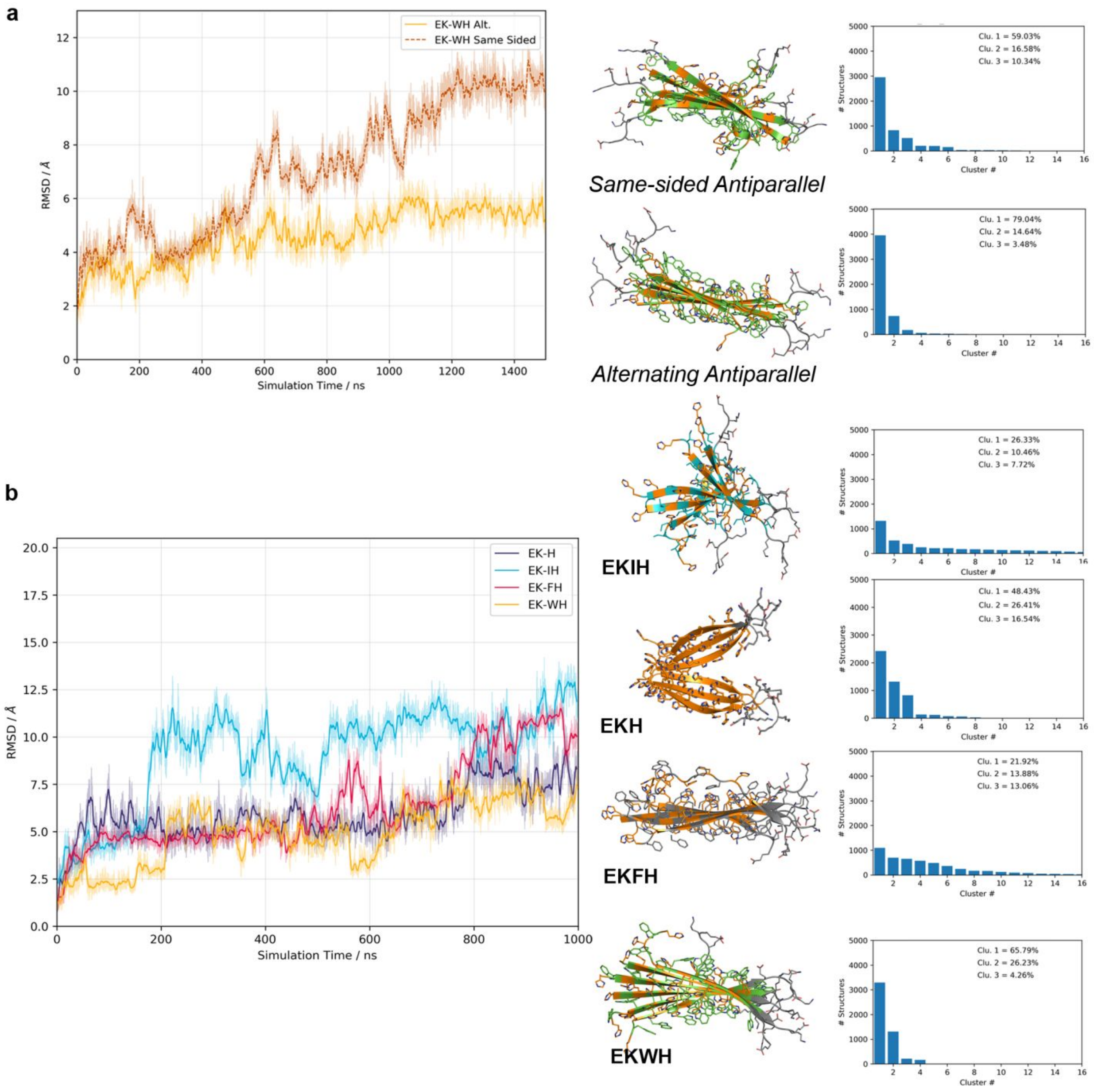

Figure S5. *Time evolution of the root-mean-square deviation (RSMD, Å) of the β-sheet structures along the MD simulations for (a) alternating (bold yellow) and same-sided (dashed brown) antiparallel arrangement. (b) RMSD for the alternating parallel β-sheets formed from EKH (purple), EKIH (cyan), EKFH (magenta), and EKWH (orange) β-strands.*

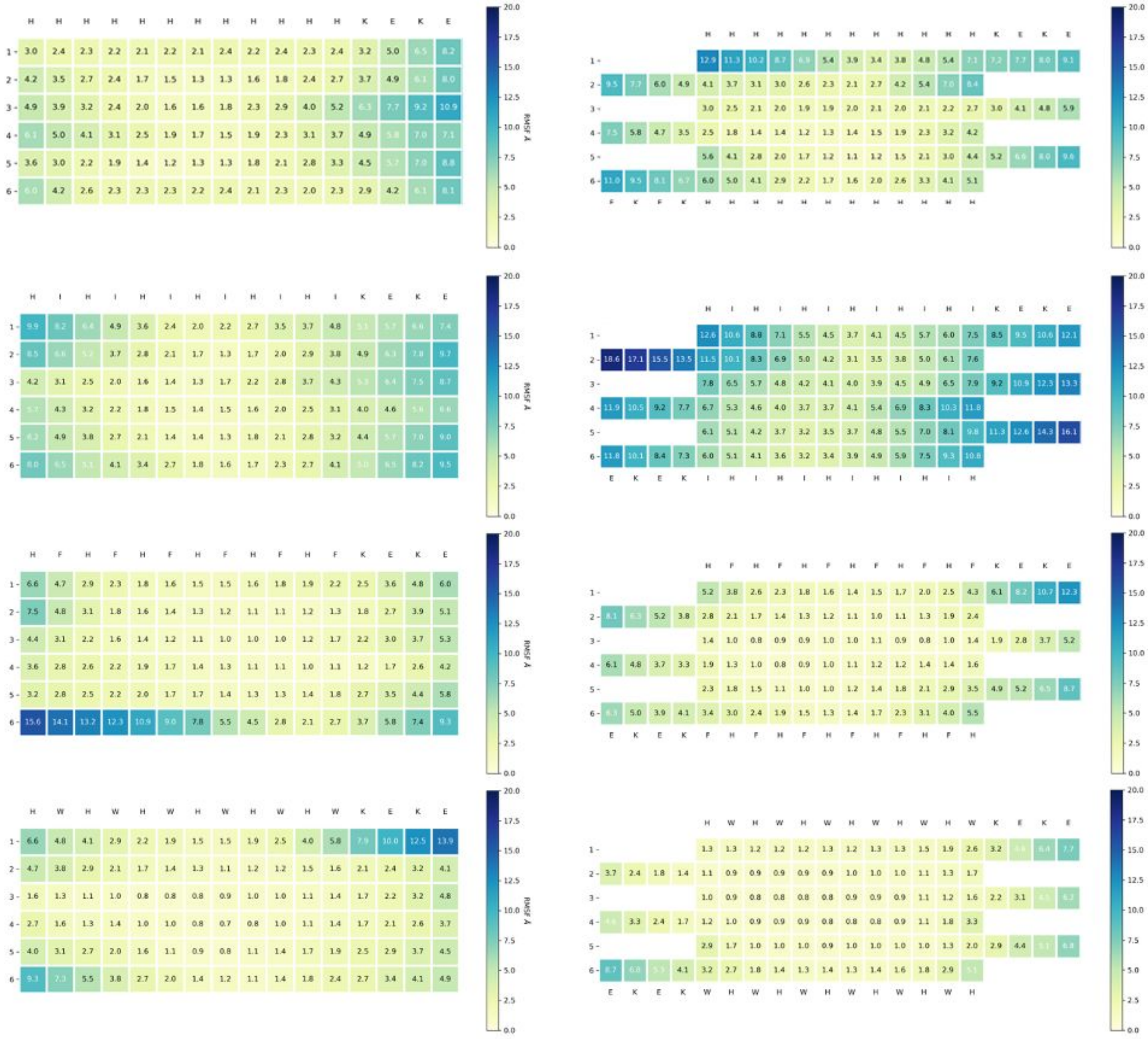

Figure S6. *Heatmaps showing the per-residue root-mean-square fluctuation (RMSF, Å) for both parallel (left) and antiparallel (right) EKH (first row), EKIH (second row), EKFH (third row) and EKWH (fourth row) β-sheets. Within each plot, each row represents one peptide – arbitrarily numbered 1-6 on the side – and the amino acid sequence is given by the one-letter code on the top. As the sequence reverses with alternating peptides within the antiparallel conformation, the flipped sequence is also given for clarity at the bottom of the plot.*

Computational results obtained for lamellar structures

Interaction Analysis

Detailed interaction analysis was undertaken, in order to better understand the difference between the EKIH and EKWH lamellar structures. The time-evolution of the RMSD (Figure S7a) indicated that EKIH evolves, during the first 400 ns, to a different metastable conformation in which it remained stabilized until the end of the simulation. A similar evolution was observed for the same-sided EKWH lamellae. However, the alternating EKWH system maintained its stability in its original conformation for the whole simulation.

To evaluate the different conformational changes that occurred within these lamellar structures, we performed a cluster analysis on the structures of the last 500 ns of the simulation (as described above). For EKIH we could distinguish up to 6 different clusters, with two significant clusters which cumulatively accounted from approximately 90% of all structures. For the EKWH same-sided arrangement, three clusters were clearly distinguished, with the most populated cluster representing around 60% of the structures of the last 500 ns. Finally, the EKWH lamellae in the alternating arrangement clearly showed only two clusters, with the first cluster representative of almost 75% of the structural conformational space for this system. This fact, taken together with the fact that only two clusters were identified for this system, further demonstrates the stability of the alternating EKWH lamellae throughout the simulation (Figure S7b).

Moreover, from a visual inspection of the systems, we observed that there were not only changes in the interactions between the layers, but also in the hydrogen bond (HB) pattern between the β-strands of each sheet, i.e., the inter-sheet interactions (Figure S7c). Following this observation, we assessed the evolution of the HB interactions between the backbone atoms of the β-strands for each β-sheet (Figure S7d). The results highlighted that the EKIH lamellae lost the initially-formed HB contacts during the first 100 ns of the simulation. This most significantly affected those β-sheets that were directly exposed to the solvent, while the β-sheets in the middle maintained a steady total number of interactions. This behavior was also observed in a more extreme fashion with the EKWH in the same-sided arrangement, where the HB network was considerably impacted in all the β-sheets. In stark contrast to this, the alternating EKWH system showed an increase in the number of HB formed over the initial 500 ns, which was then conserved until the end of the simulation. With these results, we have demonstrated that the same-sided EKWH system was the most unstable of the lamellar systems constructed. For this reason, we have followed the structural and interaction pattern analysis for only the alternating EKIH and EKWH systems.

In order to better understand these results, we have performed a contact map analysis (see above for details) of the most important interactions that changed along the simulation. We have made two contact maps, one representing the initial conformation of the system, and the other representing the final simulation output conformation. The initial conformation was selected as the output from the final equilibration step (see Computational Methods), i.e., the exact structure that started the MD simulation at t=0, and consequently is referred to henceforth as the "equilibrated" system. The second contact map was made using a conformation representative of the final result of the MD simulation, specifically the central structure of the most populated cluster from the cluster analysis of the last 500 ns (see Figure S7b). The aim of creating these two contact maps was to directly observe which internal interactions changed from the beginning of the simulation to end. We are mainly interested in the contacts that were broken and formed along the simulation, to most accurately gauge how the systems have evolved (Figure S9). These results indicate that both systems changed their initial contacts and evolve during the simulations. In the case of EKIH, this change occurred mainly at the beginning of the simulation, while for EKWH the evolution

occurred more gently and was maintained along the simulations (Figure S9b). However, this information alone does not provide an indication of how these changing interactions affected the overall structural conformation. For this reason, we have separated the broken and formed interactions by their location within the lamellar structure, that is, whether the contacts were within the same β-sheet or between the different β-sheets (see Figure S10).

From visual inspection of the equilibrated structures (Figure S10, left-most panels) we can clearly observe that for EKIH, most of the interactions were present within the β-sheets, whilst fewer interactions connected the layers, with the exception of the salt bridges formed between the Glu and Lys residues located along the edges of each β-sheet. However, for EKWH the initial contact map looks completely different. In the equilibrated structure, not only were there a lot of interactions between the residues of the same β-sheet and the salt bridges, as seen in EKIH, but also a large number between the Trp and His residues of different layers.

Furthermore, the difference in the evolution of the contact maps when comparing the final structures of EKIH and EKWH is remarkable. Although the EKWH seems to show a shift in the relative positions of the layers, the overall percentages of interactions broken (48.3%) versus those formed (51.7%) are very similar. Despite the fact that the external layers seemed to present some small instabilities, the lamellar structure as a whole was maintained and conserved. However, a completely different behavior was observed in the EKIH lamellae. Regarding the total number of interactions, only 35.5% of the measured interactions were broken versus 64.5% which were formed, a larger portion of which came from new interactions formed between the layers. Although, *a priori*, the formation of these new interactions could be considered a stabilizing effect for the whole system, when combined with a visual inspection of the final structure, it is clear that this is not the case. The β-sheets of the EKIH lamellar system underwent some form of rotation or torsion in a lateral plane, seemly creating a more a helical structure instead of a β-aggregate (see Figure S10a, middle panel).

Finally, we have further segregated these interactions that are broken and formed, both within and between the β-sheets, by the kind of non-covalent interactions due to the residues involved. We can differentiate three broad classifications of these interactions:

i. the salt bridges, formed only between Glu (E) – Lys (K) residues located at the end of the β-sheets;
ii. any of the contacts between the E and K with the hydrophobic residues of the peptides, i.e., His (H) and Ile (I) or Trp (W);
iii. and the hydrophobic contacts between the residues H-H, H-I and I-I for EKIH, or H-H, H-W and W-W for EKWH.

The pie plots in Figure S11 show the breakdown of these interaction types, with the salt bridges (i), colored blue; the mixed hydrophilic/hydrophobic contacts (ii), colored orange; and the hydrophobic-only contacts, colored green. The inter-sheet interactions (Figure S11 a-b, top) show that most of the broken interactions corresponded to those between the hydrophobic contacts, while most of the formed interactions corresponded to either contacts between the H- or W-/I- and E or (to a lesser extent) K, or E-K salt bridges. Thus, for both the EKIH and EKWH structures, the contacts that are formed heavily favor the external parts of the lamellae.

In addition to this, we would like to remark on the nature of the non-covalent interactions that are maintained along the whole simulation, which is indicative of how stable these interactions are. It is worth noting that, in this case, there are larger differences between EKIH and EKWH, mainly arising from the proportion of salt bridges that are maintained, which ranges from 4.7% for EKIH to 10.9% for EKWH.

When viewed with the number of salt bridges that are formed between the β-sheets, an even greater difference emerges between the systems. Considering that the salt bridge is a strong electrostatic non-covalent interaction, this difference could explain the higher stability of the EKWH system when compared to EKIH.

The final detail to remark on for these results concerns the formation and maintenance of the hydrophobic-only (iii) contacts. Although it appears that the EKIH forms/maintains a higher proportion than the EKWH, the nature of the specific interactions must be considered before a direct comparison can be made. This is because, whilst for EKIH it is only the H-H interaction that can make strong π-π stacking interactions, to reinforce the other hydrophobic H-I and I-I contacts, in EKWH interactions between any combination of hydrophobic residues (H-H, H-W, and W-W) can form π-π stacking interactions. Therefore, considering this disparity in the strength of the interactions it is possible to make provides further evidence for the enhanced stability of the EKWH lamellae. Altogether, these results reinforce the conclusions presented in the manuscript and help us to understand the experimental results.

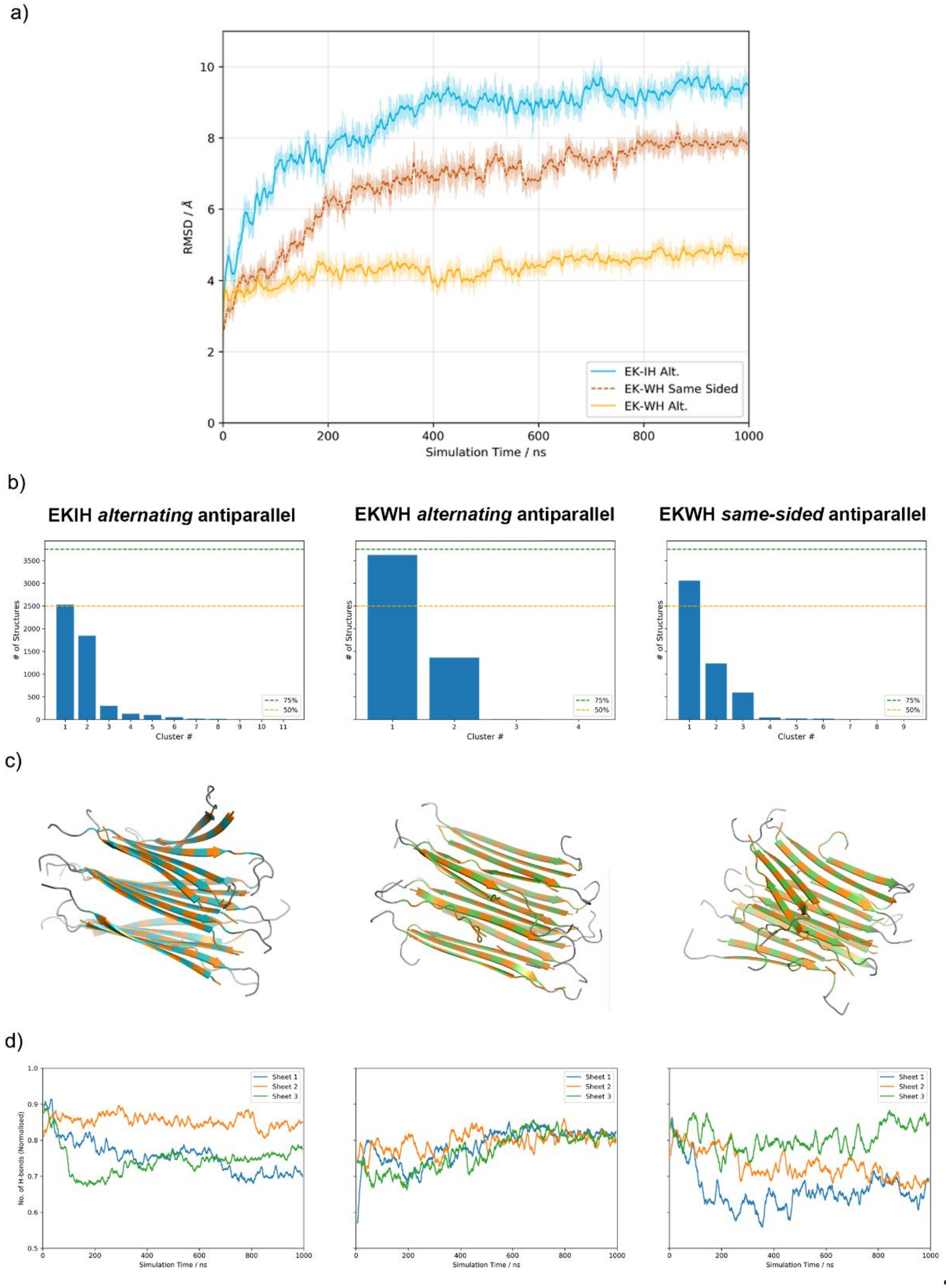

Figure S7. *(a) Time evolution of the RSMD (Å) of the lamellar structures formed by the alternating antiparallel β-sheets of EKIH (cyan) and EKWH (yellow). For the EKWH system the same-sided antiparallel β-sheets were also simulated (brown). (b) Histograms showing the results of the cluster analysis for the last 500 ns of the simulations. (c) The final snapshot of the lamellar structures for each system in a cartoon representation. (d) Time evolution of the total number of hydrogen bonds formed between the β-strands for each β-sheet of the lamellae: EKIH (left), EKWH alternating (middle) and EKWH same-sided (right) arrangement. The values plotted show a rolling average mean (with a window of 100) normalized over the maximum number of H-bonds formed per sheet per simulation.*

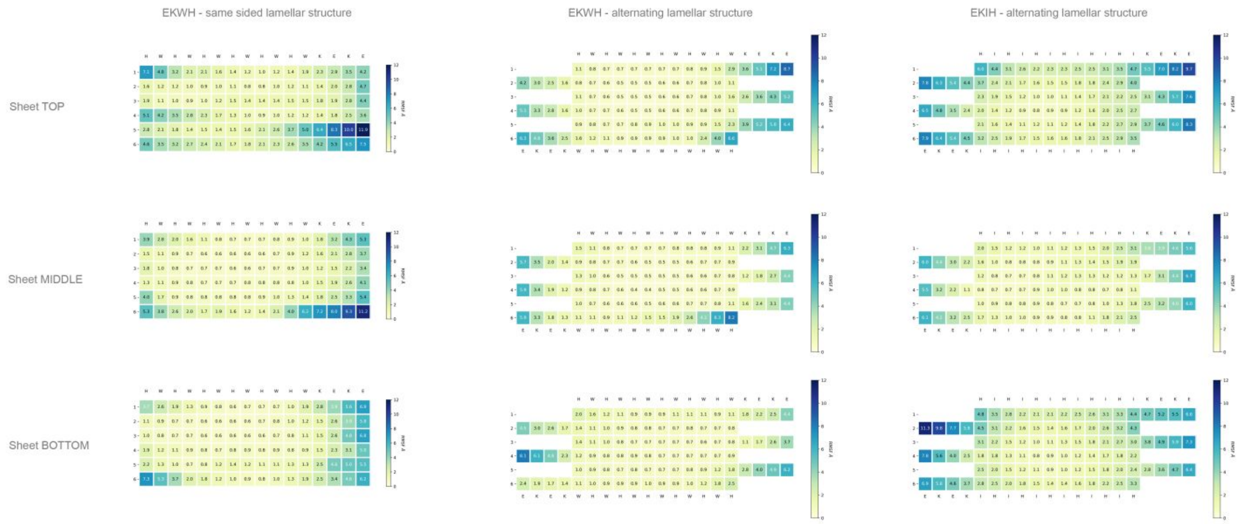

Figure S8. *Heatmaps showing the per-residue RMSF (Å) for lamellar structures simulated, EKWH same-sided antiparallel (left), EKWH alternating antiparallel (middle), and EKIH alternating antiparallel (right) β-sheets. Each row represents one peptide – arbitrarily numbered 1-6 on the side – and the amino acid sequence is given by the one-letter code on the top.*

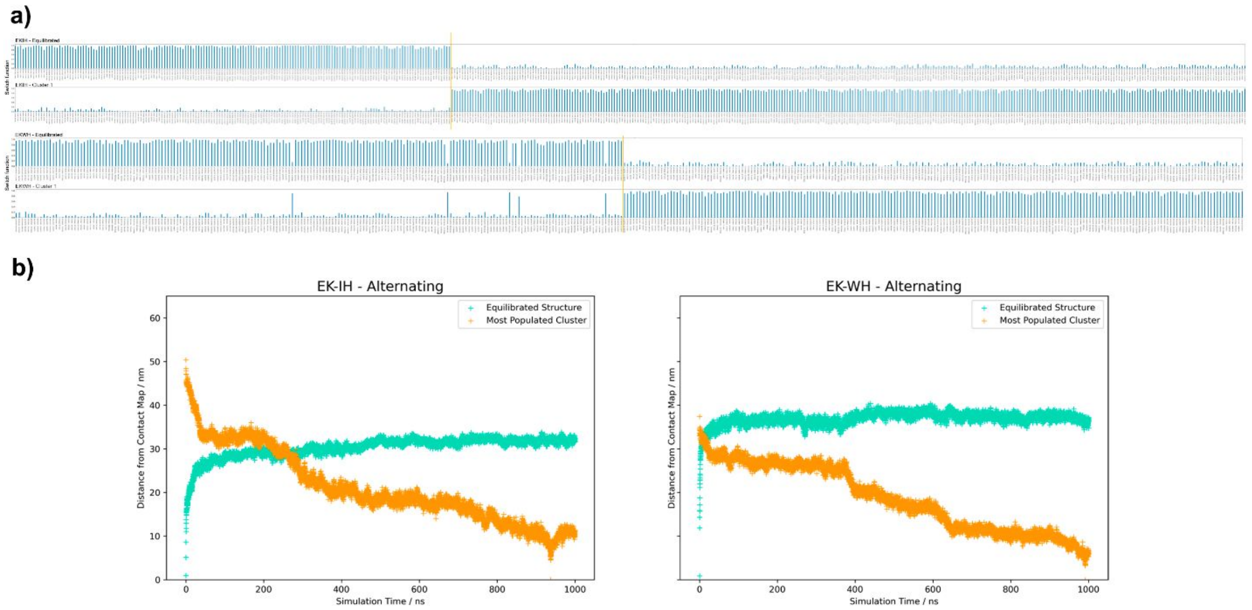

Figure S9. *(a) Contact maps of the equilibrated and clustered structures for the EKIH (top) and EKWH (bottom) systems, for which the value of the switching function changed by more than 0.75. The orange line differentiates the majority of the interactions that were found in the equilibrated system but not observed in the final clustered structure (i.e. broken contacts, left) from those that are found in the clustered structure but were not present in the equilibrated system (i.e. formed contacts, right). (b) Evolution of the simulation with respect the equilibrated (green) and the central structure of the most populated cluster (orange) contact maps for EKIH (left) and EKWH (right) systems.*

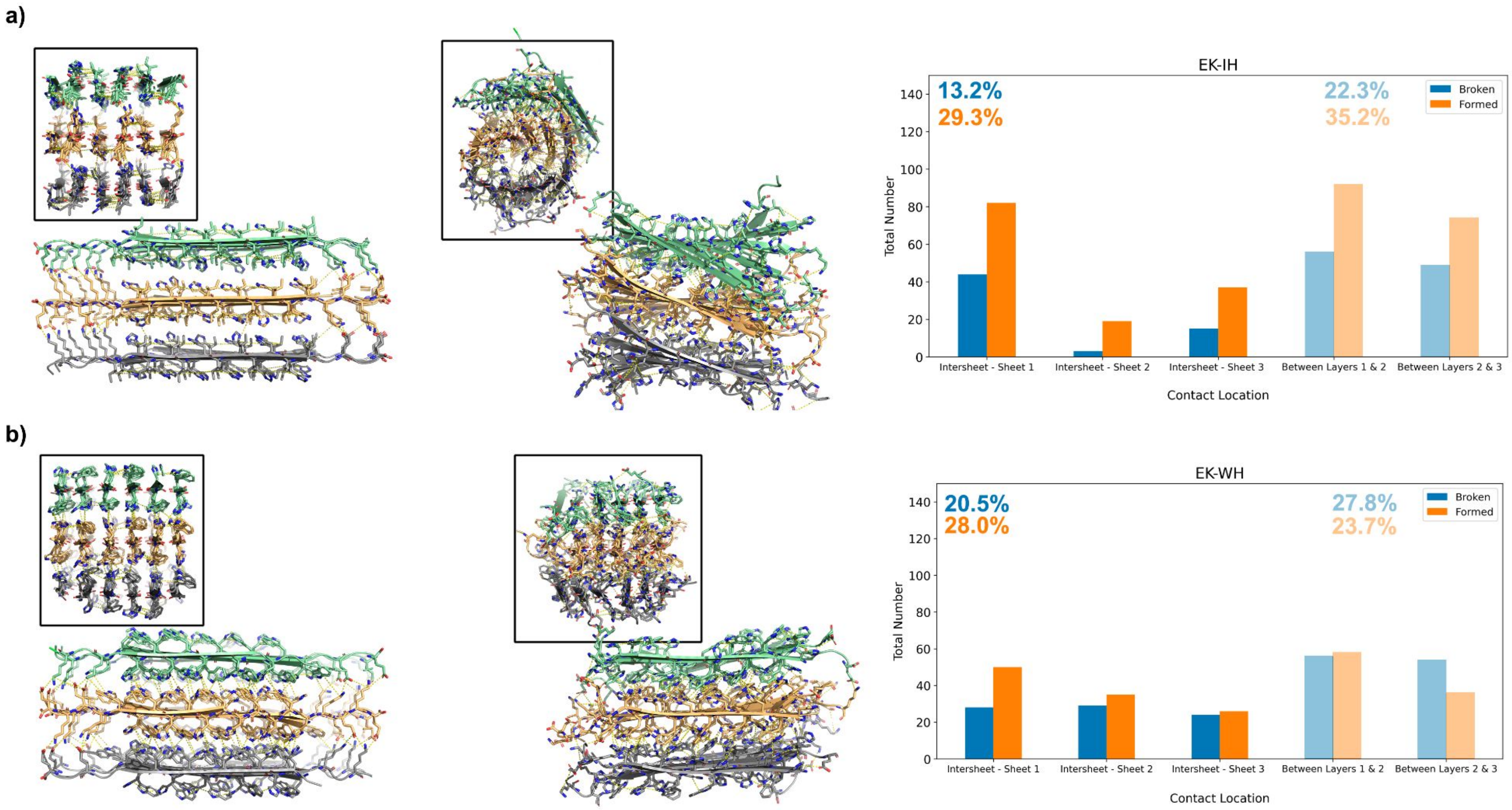

***Figure S10**. Cartoon representation of the equilibrated (left) and the central structure of the most populated cluster (middle) for the (a) EKIH and (b) EKWH lamellar systems. The inset image shows a front view of the system. The bar plots (right) show an analysis of the contacts whose switching function changed by more than 0.75 from the equilibrated contact map to the final contact map, categorized by the location of, and change exhibited by, each contact. The total % of interactions **formed** within the same β-sheet (orange) and between different β-sheets (light orange) and the total % of interactions **broken** within the same β-sheet (blue) and between different β-sheets (light blue) are also given for each plot.*

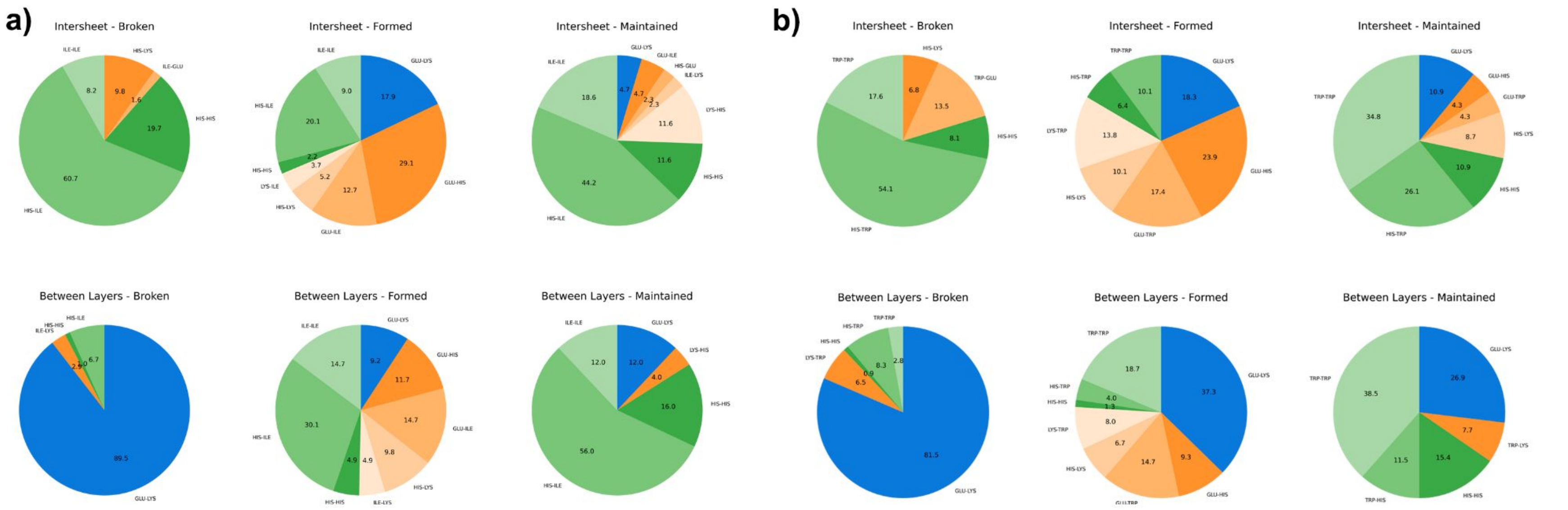

Figure S11. *Pie charts indicating the residue-residue contacts that are broken, formed, and maintained within the β-sheets (intersheet, top row) and between the β-sheets (bottom row) for the (a) EKIH and (b) EKWH lamellar systems. The colors of the segments indicate the nature of non-covalent interaction for each residue pair, with blue for the salt bridge between Glu– Lys residues, located at the edges of the β-sheets; oranges for the interactions between the hydrophilic and the hydrophobic residues of the peptides, i.e., Glu or Lys with His or Ile/Trp; and greens for the hydrophobic-only contacts, between residues His and Ile or Trp.*
